# Operative versus conservative treatment for patellar dislocation: a meta-analysis of 7 randomized controlled trials

**DOI:** 10.1186/1746-1596-9-60

**Published:** 2014-03-18

**Authors:** Biao Cheng, Xing Wu, Heng’an Ge, Ye qing Sun, Qiang Zhang

**Affiliations:** 1 Department of Orthopedics, Shanghai Tenth People's Hospital, Tongji University School of Medicine, 301 Yanchang Middle Road, Shanghai 200072, China; 2 First Clinical Medical College, Nanjing Medical University, Nanjing, China

## Abstract

**Purpose:**

Patellofemoral pathology is common, and patellofemoral dislocation mainly affects adolescents and young adults. We conducted a meta-analysis exclusively of RCTs to compare the clinical outcomes of patellar dislocation patients managed operatively versus non-operatively.

**Methods:**

After systematic review of electronic databases and websites, a total of 7 RCTs reporting data on 402 subjects were included. The methodological quality of the literature was assessed using the PEDro critical appraisal tool. Mean differences (MDs) and risk ratio (RR) were calculated for the pooled effects. Heterogeneity was assessed using the I^2^ test.

**Results:**

Data synthesis showed a lower rate of recurrent patellar dislocation post-treatment in patients managed operatively compared to non-operatively (*P* = 0.01).

**Conclusion:**

The results suggest a difference in outcomes between the treatment strategies. However the limited number of studies and high risk of inherent bias indicate that future studies involving more patients in better-designed randomized controlled trials will be required.

**Virtual slides:**

The virtual slide(s) for this article can be found here: http://www.diagnosticpathology.diagnomx.eu/vs/8011948721221355.

## Introduction

Patellofemoral pathology is one of the most common conditions in the clinical work of the general orthopedist, with patellofemoral dislocation mainly affecting adolescents and young adults [1,2]. Patellar dislocation accounts for 2–3% of all knee lesions and is the second most common cause of traumatic haemarthrosis of the knee [3,4]. There are various anatomical factors which predispose individuals to patellar dislocation, such as trochlear dysplasia, abnormal extensor mechanism alignment, patella alta, hypermobility syndrome, a tight lateral retinaculum, hypoplasia of the vastus medialis oblique (VMO), occurrence in youth, family history, and bilateral symptoms [5-7].

Proper treatment is essential in order to minimize sequelae such as recurrent dislocation, painful subluxation, and osteoarthritis [8]. Traditionally, patients have been managed non-operatively following a first time dislocation, except when associated patellar displacement or osteochondral fractures of the lateral femoral condyle are present [7,9-11]. This non-operative strategy often consists of a period of immobilization in a splint or cast, followed by physiotherapy, principally of the quadriceps complex [12-14]. However, the literature has suggested that the recurring instability rate is more than 50% after non-operative treatment and thus some authors have advocated that surgical intervention such as repair or reconstruction of the medial retinaculum and medial patellofemoral ligament (MPFL), medialisation of the tibial tubercle, and lateral release procedures should be performed [15-22].

To date, there have been seven prospective randomized trials and one meta-analysis comparing conservative and operative treatment after patellar dislocation. The meta-analysis including 11 publications (only five randomized controlled trials (RCTs)) indicated lower redislocation rates, but higher rates of patellofemoral osteoarthritis after operative treatment [6]. The author of that analysis considered that this finding should be interpreted with great caution, since the inclusion of too many non-RCTs is the weakness of that study. Therefore, it is necessary to conduct a meta-analysis including only RCTs to compare the clinical outcomes of patients managed operatively compared to non-operatively following a patellar dislocation.

## Method

### Study sources and searches

The literature search was conducted in Medline, PubMed, Embase, and The Cochrane Library Central register of controlled trials to identify relevant published English articles from January 1966 to December 2013. The search key words and subject terms used were “patellar dislocation” “patella” “patellar subluxation” and “patellar instability”. Relevant articles in reference lists of published articles were also searched.

### Study selection and data extraction

All studies that were identified by the literature searches were reviewed and selected according to the following prior criteria: (i) patients with patellar dislocation regardless of age and sex; (ii) RCTs with two groups comparing operation with conservative treatment; and (iii) outcomes of operative or non-operative management of patients. Authors of selected studies were contacted for further information when necessary.

Data was extracted by two independent reviewers. The extracted information included: (i) the first author, year of publication, study type and study duration; (ii) the number and characteristics of subjects; (iii) operative interventions undertaken, non-operative strategies and treatment duration; and (iv) outcomes. The two reviewers reached agreement on selected articles and extracted information and if they disagreed, a third reviewer was invited to resolve the differences. In cases of missing data, or when mean or standard deviation (SD) values were not presented, corresponding authors were contacted to attempt to obtain this data. Once completed, all data were then synthesized into an agreed data extraction table. This formed the basis of the results for data analysis.

### Evaluation of methodological quality

Data were extracted by one main researcher and then verified by another researcher. Any discrepancies were resolved by discussion. The methodological quality of each study was assessed using the Physiotherapy Evidence Database (PEDro) scale [23]. To minimize selection bias, two investigators rated each study independently and subsequently assigned a score based on the PEDro scale.

### Outcome measurement

The frequency of recurrent patellar dislocation was used as the primary outcome in patients managed operatively compared to non-operatively following a patellar dislocation under investigation. Secondary outcomes under investigation included functional outcomes assessed using the Kujala score [24], Tegner activity score [25], pain, frequency of recurrent instability, Hughston visual analog score (VAS) [26], patient satisfaction, return to functional activities, and frequency of subsequent surgical intervention.

### Statistical analysis

All statistical analyses were performed using Review Manager 5.2. Analysis of the treatment effect was performed when no substantial differences in study populations, interventions or outcome measurements were observed. The chi-squared statistic and the I^2^ statistic were used to assess heterogeneity. Studies with an I^2^ statistic of >75% were considered to have a high degree of heterogeneity; studies with an I^2^ statistic of 50–75% were considered to have a moderate degree of heterogeneity; and studies with an I^2^ statistic of 25–50% were considered to have a low degree of heterogeneity. Publication bias was not examined due to the small number of studies (< 10) included in each analysis.

A fixed-effect model was initially employed in the analysis, unless significant heterogeneity was observed; a random effects model analysis was used in order to account for the extra uncertainty due to heterogeneity. For continuous outcomes with the same measurement scale, means were computed with 95% confidence intervals (CIs). However, for continuous outcomes with different measurement scales, standardized mean difference (SMD) was calculated. The dichotomous outcomes were presented as a risk ratio (RR) with 95% CIs. A *P* value lower than 0.05 or a 95% CI that did not contain unity was considered statistically significant. Outcomes were summarized and expressed using a forest plot. Descriptive analysis was used for any individual result which was not available for meta-analysis.

## Result

### Search results

**Figure 1:**
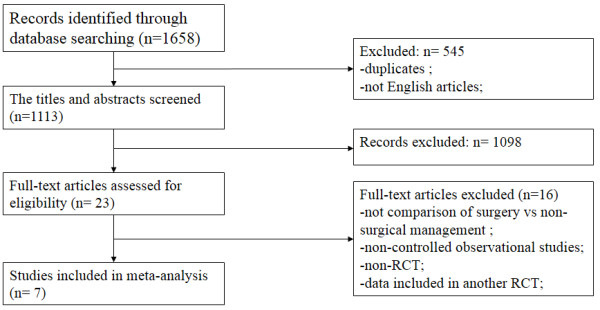
Flow diagram of study selection.

**Figure 2:**
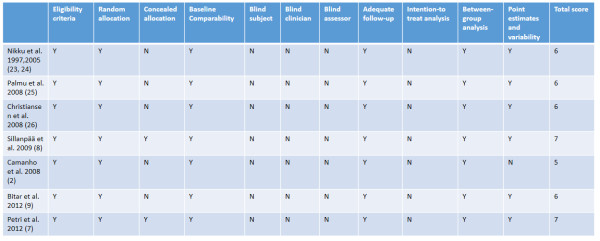
PEDro critical appraisal score.

Eight studies [2,7-9,27-30] were included in this meta-analysis with a total of 402 patients. The flow diagram of the study search process is presented in Figure 1. We identified two published papers describing a single RCT [27,28]. The characteristics of the included studies are provided in Table 1. The characteristics of patients in the included studies are listed in Table 2. In cases of conservative treatment, the position and duration of immobilization, and physiotherapy regimes, were noted. In respect to operative intervention, lateral release or MPFL repair were performed in the studies. The mean PEDro score of the 7 trials was 6.0 (SD = 0.63), and detailed results are summarized in Figure 2. Blinded subjects, blinded clinicians and intention-to-treat analysis were not used in any of the RCTs. Only two of the seven RCTs used the concealed allocation method. One RCT did not report the point estimates or variability.

**Table 1:** Characteristics of studies included in this meta-analysis

**Study**	**National**	**Study design**	**Operative interventions**	**Non-operative interventions**	**Duration**
Nikku et al. 1997, 2005 [23,24];	Japan	RCT	MR or MPFL augmentation, or LR.	3 weeks immobilization in cast or orthosis then thigh muscle exercises	7.2y
Palmu et al. 2008 [25];	Finland	RCT	MPFL repair and in 32 cases, LR.	3 weeks immobilization in cast or orthosis then thigh muscle exercises.	14y
Christiansen et al. 2008 [26];	Denmark	RCT	MPFL repair	0–2 weeks orthosis immobilization 0–20° motion. Quadriceps exercises and general physiotherapy	2y
Sillanpää et al. 2008 [8];	Finland	RCT	MR and MPFL repair; R-G procedure; arthroscopic repair of osteochondral fracture	3 weeks orthosis immobilization 0–30° motion. Week 3–6 weeks, 0–90° full motion at 6 weeks. Quadriceps exercises commence immediately	7y
Camanho et al. 2008 [2];	Brazil	RCT	MPFL repair	3 weeks immobilization then physiotherapy with VMO exercises	Op-3.4; N-Op 3.0
Bitar et al. 2012 [9];	Brazil	RCT	MPFL reconstruction using patellar tendon	3 weeks immobilization then physiotherapy with quadriceps exercises	2y
Petri et al. 2012 [7];	Germany	RCT	repair the tear, or lateral release	3 weeks orthosis immobilization 0–30° motion with partial weight-bearing. Week 3–6 weeks, 0–90° motion with progression to pain-adapted full weight-bearing.	2y

MR: Medial retinaculum; MPFL: Medial patellofemoral ligament; LR: Lateral release; Op: Operative; R-G: Roux–Goldthwaite procedure; RCT: Randomized controlled trial; N-op: Non-operative; VMO: Vastus medialis oblique.

**Table 2:** The patient characteristics of the included studies

	**Sample size**		**Mean age**		**Gender(M/F)**	
	**Op**	**N-op**	**Op**	**N-op**	**Op**	**N-op**
Nikku et al. 1997, 2005 [23,24];	70	57	20	20	18/52	27/30
Palmu et al. 2008 [25];	36	28	13	13	9/27	9/19
Christiansen et al. 2008 [26];	42	35	20	19.9	24/18	18/17
Sillanpää et al. 2008 [8];	18	22	20	20	17/1	20/2
Camanho et al. 2008 [2];	17	16	24.6	26.8	6/11	7/9
Bitar et al. 2012 [9];	21	20	23.9	24.1	9/12	11/9
Petri et al. 2012 [7];	12	8	27.2	21.6	8/4	5/3

M: Male; F: Female; Op: Operative; N-op: Non-operative.

### Results of pooled analysis

**Figure 3:**
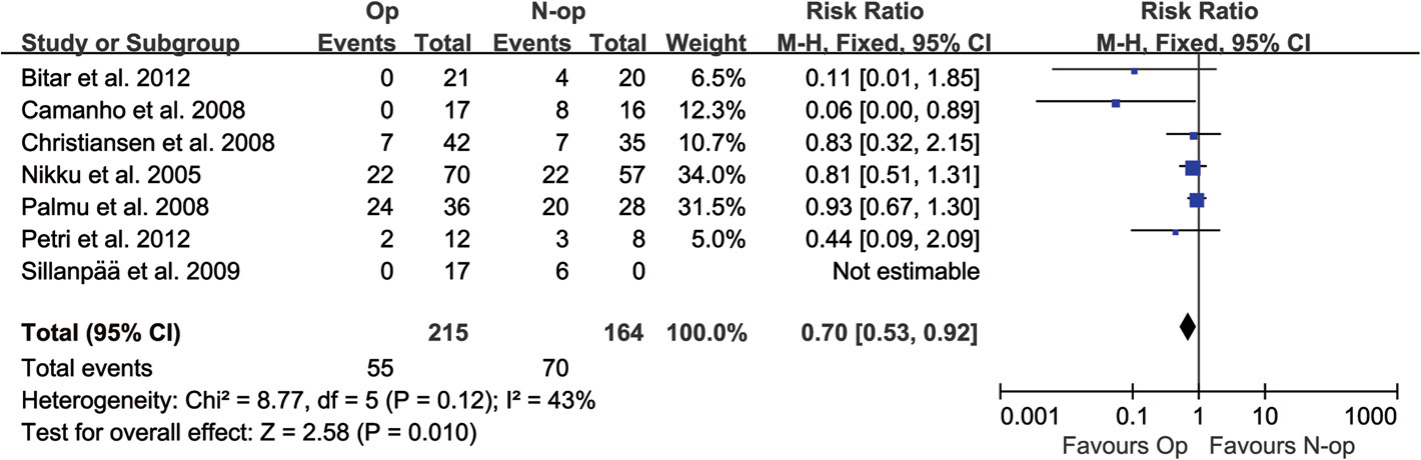
Forest plot to assess recurrent dislocation events between two treatment strategies.

The detailed results of the pooled analysis are presented in Table 3. As the study’s primary outcome, analysis using a fixed effect model revealed a statistically significant difference between the two treatment groups in respect to recurrent patellar dislocation (*P* = 0.01, 95% CI: 0.53–0.92, *I*^2^ = 43%) (Figure 3). A higher rate of recurrent patellar dislocation events post-treatment was demonstrated in patients managed non-operatively compared to those managed operatively.

**Table 3:** The results of pooled analysis

**Outcome**	**Study**	**Risk ratio effect/Mean difference(95%CI)**		***P *** **Value**	**Heterogeneity**	
		**Random effects**	**Fixed effects**		***I***^**2**^	***P *** **Value**
Frequency of recurrent dislocation	[2,7-9,23-26]	0.73 [0.46, 1.16]	0.70 [0.53, 0.92]	0.01	43%	0.12
Frequency of recurrent instability/subluxation	[2,7-9,23,24]	0.67 [0.25, 1.80]	0.98 [0.75, 1.28]	0.86	57%	0.07
Patient satisfaction (excellent/good)	[7,23-25]	0.84 [0.71, 1.00]	0.84 [0.70, 1.00]	0.05	0	0.93
Frequency subsequent surgery required	[8,25]	0.74 [0.15, 3.70]	0.94 [0.53, 1.66]	0.82	41%	0.19
Kujala score	[2,7-9,23-26]	6.38 [-5.32, 18.08]	9.40 [8.18, 10.61]	0.29	98%	<0.00001
Hughston VAS	[23-25]	-5.68 [-9.49, -1.88]	-5.68 [-9.49, -1.88]	0.003	0	0.66
Tegner score	[8,23-25]	-1.09 [-1.54, -0.63]	-1.02 [-1.19, -0.85]	<0.00001	28%	0.25

**Figure 4:**
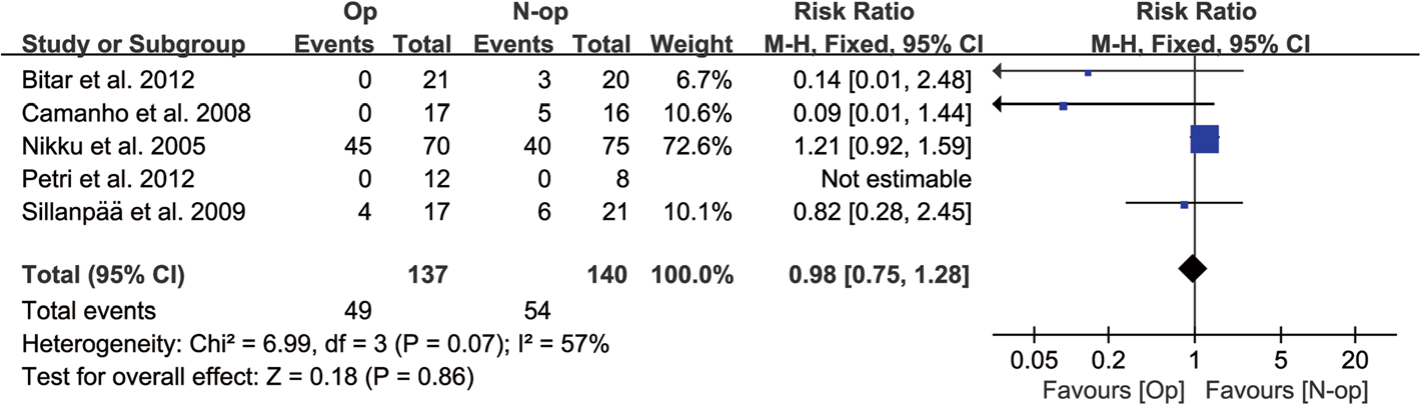
Forest plot to assess frequency of recurrent instability/subluxation events between two treatment strategies.

Data on frequency of recurrent instability/subluxation rate was available in all RCTs [2,7-9,28-30]. After meta-analysis, no significant effect of the frequency of recurrent instability/subluxation was observed (RR = 0.82, 95% CI: 0.75–1.28, *P* = 0.86), with a moderate degree of heterogeneity (*P* = 0.07, *I*^2^ = 57%) (Figure 4).

**Figure 5:**
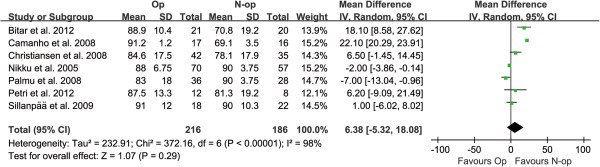
Forest plot to assess Kujala scores between two treatment strategies.

After meta-analysis of all included RCTs [2,7-9,28-30], no significant difference in the Kujala score between the two treatment groups was observed (MD = 6.38, 95% CI: -5.32–18.08, *P* = 0.29), with a high degree of heterogeneity across the studies (*P* < 0.00001, *I*^2^ = 98%) (Figure 5).

**Figure 6:**
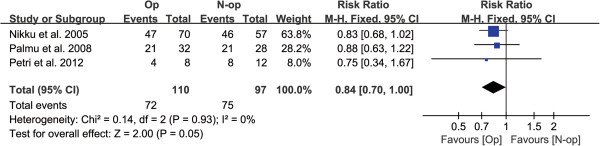
Forest plot to assess patient satisfaction between two treatment strategies.

With respect to patient satisfaction or frequency of the requirement for subsequent surgery, a statistically significant difference was found between the two treatment groups (*P* = 0.05, Table 3, Figure 6), without significant heterogeneity.

**Figure 7:**
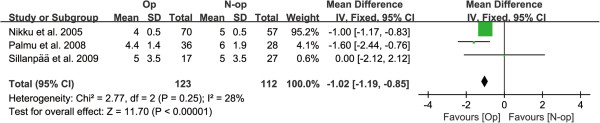
Forest plot to assess Tegner scores between two treatment strategies.

**Figure 8:**
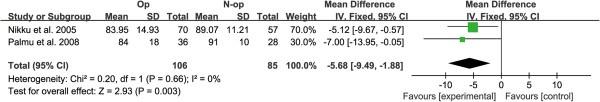
Forest plot to assess Hughston VAS scores between two treatment strategies.

After combining the data from three of the seven included RCTs [8,27-29], a significantly higher Tegner score was observed in the non-operative group compared to patients who received operative management (MD = -1.02, 95% CI: -1.19 to -0.85, *P* < 0.00001), with a low degree of heterogeneity across the studies (*P* = 0.25, *I*^2^ = 28%) (Figure 7). After meta-analysis of two RCTs [27-29], a significantly better outcome in Hughston VAS score with higher scores was observed in the non-operative group, compared to those who received operative management (MD = -5.68, 95% CI: -9.49 to -1.88, *P* = 0.003), without significant heterogeneity across the studies (*P* = 0.66, *I*^2^ = 0) (Figure 8).

## Discussion

In this meta-analysis, we summarized findings in the clinical literature on the outcomes of operative versus conservative treatment for patellar dislocation. On the basis of the available evidence, this study demonstrates a higher rate of recurrent patellar dislocation events post-treatment, higher Tegner score and higher Hughston VAS score in patients managed non-operatively compared to patients managed operatively.

All RCTs [2,7-9,28-30] suggested that operative treatment of patellar dislocation results in a lower risk of recurrent patellar dislocation compared to non-operative treatment. There were six RCTs in which patients were managed operatively compared to non-operatively following the first patellar dislocation among the seven included RCTs. Therefore, the finding should be interpreted with great caution with reference to recurrent patellar dislocation. In 2011, Smith *et al*. showed equivalent results in their meta-analysis [6]. They considered that the result should be interpreted with great caution due to statistically significant funnel plot asymmetry. In a study by Palmu et al. [29], although the rate of recurrent patellar dislocation was higher in patients following non-operative treatment, there was no significant difference in functional assessment. This suggested that these patients were able to perform all their activities of daily living, irrespective of recurrent patellar instability and dislocation events. Therefore, assessment of functional-based outcomes should be paid more attention in future studies.

This study reported that there was a difference in Hughston VAS score when comparing pain between operative and non-operative management strategies. The degree of pain was lower in the non-operative group. However, the finding should be interpreted with great caution because the data involved in this result was extracted from only two RCTs and the Kujala score [2,7-9,28-30] correlated better with the subjective result and the recurrence of patellar dislocation than the Hughston VAS score [24]. There was no significant difference in Kujala score between the two treatment groups (MD = 6.38, 95% CI: -5.32 to 18.08, *P* = 0.29), with a high degree of heterogeneity across the studies (*P* < 0.00001, *I*^2^ = 98%) in this analysis. The reasons for this result may include the following factors. First, the Kujala score is a subjective evaluation method so that there may have been differences between the included RCTs. Second, the inclusion criteria were different for each study included. Third, there was some variability in treatment methods, especially in operative treatment. The seven RCTs reported using a number of different operative interventions including lateral release, medial retinaculum or MPFL repair, or Roux–Goldthwaite procedures.

Only one study was identified which solely assessed the incidence of patellofemoral osteoarthritis between operative and non-operative groups. [8] This reported that there was no statistically significant difference between patients treated operatively and those treated non-operatively in respect to articular cartilage lesions within the patellofemoral joint. Repeated chondral injury may predispose patients to osteoarthritis [31-33]. Due to the relative scarcity of RCTs assessing the incidence of patellofemoral osteoarthritis in patients managed following patellar dislocation, it will be necessary to observe whether there is a difference in clinical outcomes between patients managed non-operatively and operat'ively.

The results of this review should be interpreted and generalized with caution due to the limited number of the studies and the high risk of bias inherent in the studies. First, it included only a limited number of studies and of subjects. After a careful search, only 7 RCTs were included in the final analysis, giving a total population of 402 subjects. The publication bias was not tested in our analysis in consideration of the low power due to the small number of studies included.

Second, of the seven RCTs, only two used an appropriate concealed allocation method for randomization, and none reported adequate intention-to treat analysis. This might have introduced selection bias. In addition, blinding of outcome assessors was not used in any of the RCTs included, and thus detection bias might have been introduced.

Third, most of the studies did not screen participants for stress and anxiety levels, which might have weakened the evidence of the study. Four, while operative and non-operative interventions were compared in those RCTs included, the majority of studies poorly described the specific management procedures in detail, therefore limiting the ability to replicate these clinical trials. In particular, the non-operative management strategies were poorly described in all RCTs.

Finally, as regards the functional outcome of patients following patellar dislocation, there was no difference in Kujala score between operative and non-operative management strategies with significant heterogeneity. This subjective result was not effectively evaluated.

In future, in order to better evaluate the outcomes of the two treatment strategies, it is suggested to define the population, standardize the interventions prescribed to those patients, and evaluate this area of therapy through a well-designed randomised controlled trial.

## Conclusions

The current systematic review with meta-analysis demonstrated a lower rate of recurrent patellar dislocation events post-treatment in patients managed operatively compared to patients managed non-operatively. Due to the limited number of the studies available, the findings of this study should be interpreted with caution. Further RCTs including a larger number of patients and a better-designed controlled trial are desirable in future.

## Competing interests

The authors declare that they have no competing interests.

## Authors’ contribution

BC and XW carried out the search of the literatures, extracted the data and drafted the manuscript. HG performed the Evaluation of methodological quality. YQS and QZ performed the database setup and statistical analysis. QZ helped to draft the manuscript. All authors read and approved the final manuscript.

## Authors’ information

Biao Cheng and Xing Wu are co-first authors.
